# Anaesthetic Management Including Low-Dose Dexmedetomidine and Butorphanol in Dogs Undergoing ECG-Gated Cardiac Computed Tomography: A Retrospective Case Series †

**DOI:** 10.3390/vetsci13080767

**Published:** 2026-07-31

**Authors:** Camilla Schiesari, Luigi Tucci, Arianna Costa, Laura Ventura, Giovanna Bertolini, Elisa Bortolami

**Affiliations:** 1 Anesthesia and Analgesia Division, San Marco Veterinary Clinic and Laboratory, Veggiano, 35030 Padova, Italy; camilla.schiesari@vetagro-sup.fr (C.S.);; 2 Diagnostic and Interventional Radiology Division, San Marco Veterinary Clinic and Laboratory, Veggiano, 35030 Padova, Italybertolini@sanmarcovet.it (G.B.); 3 Department of Statistical Sciences, Padua University, 35121 Padua, Italy; ventura@stat.unipd.it

**Keywords:** dexmedetomidine, anaesthesia, dogs, cardiac computed tomography, ECG-gated CT, cardiac tamponade, pericardial effusion

## Abstract

This retrospective case series aims to assess the effects of an anaesthetic protocol including low-dose dexmedetomidine and butorphanol in dogs referred for cardiac tamponade undergoing ECG-gated CCT with contrast medium administration. Fifteen dogs (7 males, 8 females; ASA III/IV, body weight 25 ± 17.5 kg and age 8.6 ± 3.2 years) of different breeds with mild (*n* = 10/15) and moderate (*n* = 5/15) pericardial effusion were included. Pericardiocentesis was performed before anaesthesia. Dogs were premedicated with intravenous (IV) dexmedetomidine (0.5–1 μg kg^−1^) and butorphanol (0.1–0.3 mg kg^−1^). General anaesthesia was induced with propofol to effect (2–4.5 mg kg^−1^) IV and maintained with isoflurane in medical air and oxygen. During anaesthesia, heart rate and mean arterial pressure ranged from 37–141 beats min^−1^ and 58–117 mmHg respectively. No arrhythmias were recorded; mild hypotension occurred in two patients under general anaesthesia. No mortality was recorded within 48 h. Image quality was rated as excellent or good in most examinations, with moderate quality reported in two cases. A protocol including low-dose dexmedetomidine and butorphanol was feasible and was associated with acceptable recorded cardiovascular variables in this case series.

## Simple Summary

Cardiac computed tomography is used to evaluate heart disease in dogs, but general anaesthesia is usually required to obtain images of adequate quality. This retrospective study describes the use of an anaesthetic protocol including low-dose dexmedetomidine and butorphanol in fifteen client-owned dogs presenting with cardiac tamponade undergoing cardiac computed tomography with contrast administration. All dogs underwent pericardiocentesis before induction of general anaesthesia, with the timing determined by their clinical condition. Cardiovascular variables remained within acceptable ranges in most patients, with mild reductions in blood pressure observed in two dogs and no abnormal heart rhythms recorded. All examinations were completed, image quality was adequate for diagnostic interpretation, and no deaths occurred within forty-eight hours.

## 1. Introduction

Cardiac computed tomography (CCT) represents an advanced diagnostic procedure that enables detailed visualization of the heart and major vascular structures, thereby facilitating the identification of congenital and acquired cardiac disorders in canine patients [1,2,3]. However, in veterinary species, pharmacological restraint is generally required in order to ensure adequate image quality and to avoid motion artefacts. Furthermore, general anaesthesia and controlled mechanical ventilation are recommended since apnoea is often required to minimise respiratory artifacts responsible for the substantial degradation of image quality [4].

General anaesthesia in dogs with pre-existing cardiovascular disease poses unique challenges, as commonly utilized anaesthetic agents have the potential to depress cardiac function, thereby exacerbating hemodynamic instability and leading to complications such as hypotension, arrhythmias, and other adverse events [5]. These risks underline the importance of careful patient selection and the implementation of tailored anaesthetic protocols that prioritize cardiovascular stability. Dexmedetomidine, an α2-adrenergic agonist, is widely used in veterinary anaesthesia for its sedative and analgesic effects [6]. However, its use in cardiopathic dogs remains controversial due to its dose-dependent effects on heart rate and blood pressure, including bradycardia and peripheral vasoconstriction [6].

In humans, CCT is usually performed in conscious patients. Sometimes sedation may be used particularly in paediatric populations or in non-cooperative patients [7,8]. Dexmedetomidine has been increasingly employed in this setting because it provides reliable sedation with minimal respiratory depression while preserving spontaneous ventilation [9]. Although reductions in heart rate and variations in arterial blood pressure are commonly observed, these effects are generally predictable and dose-dependent [6,7]. Beyond its sedative profile, dexmedetomidine has been investigated for its potential cardioprotective properties in human medicine. Clinical and experimental evidence suggests that attenuation of sympathetic tone and reduction in myocardial oxygen demand may contribute to modulation of perioperative cardiovascular stress responses, particularly in cardiac surgical contexts [10]. Experimental studies have further indicated that dexmedetomidine may exert cardioprotective effects, potentially mediated by anti-inflammatory mechanisms and modulation of sympathetic activity [11].

In veterinary literature, evidence regarding the cardiovascular effects of dexmedetomidine is largely derived from experimental studies conducted in anesthetised healthy animals [12]. In these settings, dexmedetomidine has been shown to induce dose-dependent reductions in heart rate and changes in haemodynamic parameters. Echocardiographic investigations have demonstrated transient alterations in cardiac dimensions and systolic indices following administration [13]. Experimental data have also shown dose-related modulation of myocardial oxygen consumption and coronary vascular tone [14]. However, data from clinical populations of cardiopathic dogs remain limited. A retrospective study evaluating dexmedetomidine as an adjuvant to general anaesthesia during pulmonic balloon valvuloplasty reported acceptable haemodynamic stability and a reduced requirement for vasoactive and antimuscarinic drugs, suggesting that its use may be feasible in selected cardiopathic patients [15].

This study aims to retrospectively describe the feasibility of an anaesthetic protocol including low-dose dexmedetomidine and butorphanol in dogs presenting with cardiac tamponade undergoing ECG-gated CCT, and to expand the limited body of literature on anaesthetic protocols for cardiovascular imaging in veterinary practice.

## 2. Materials and Methods

### 2.1. Study Design and Population

This was a retrospective observational study based on the review of anaesthetic records of client-owned dogs referred to the San Marco Veterinary Clinic and Laboratory between February 2021 and May 2024 for cardiac tamponade and undergoing contrast-enhanced, ECG-gated cardiac computed tomography.

All procedures were performed exclusively for diagnostic and clinical purposes and for the benefit of the patients. No procedures were performed for research purposes. Written informed consent for the diagnostic and anaesthetic procedures was obtained from all dog owners. The retrospective use of anonymised clinical data was reviewed and approved by the Institutional Committee of the San Marco Veterinary Clinic and Laboratory.

Inclusion criteria were: sedation with dexmedetomidine and butorphanol, induction of anaesthesia with propofol and maintenance of anaesthesia with isoflurane. Exclusion criteria included absence of pericardial effusion and use of other anaesthetic protocols. All patients underwent a comprehensive clinical examination prior to the procedure, blood tests (complete blood count, biochemical and coagulation profiles) and urinalysis; vital parameters, including heart rate (HR), respiratory rate (RR), temperature, and systemic arterial blood pressure, were measured and recorded using a veterinary-specific non-invasive blood pressure (NIBP) monitor (Vet30, SunTech Medical, Inc., Morrisville, NC, USA), which has been validated in accordance with the guidelines of the American Animal Hospital Association (AAHA) [16,17]. Additional diagnostic evaluations included thoracic radiography, echocardiography, and electrocardiography in a fully conscious state, performed by a board-certified veterinary cardiologist. All dogs presented with cardiac tamponade and were classified according to the American Society of Anaesthesiologists physical status classification system [18]. Ultrasound-guided pericardiocentesis prior to induction was performed in all the animals.

### 2.2. Anaesthetic Protocol

All animals were submitted to a period of fasting of 8–12 h, but had free access to water before anaesthesia [17]. One over-the-needle catheter (Introcan Safety^®^, B. Braun Melsungen AG, Melsungen, Germany) was aseptically placed in a cephalic vein, and isotonic crystalloid fluid therapy using Ringer’s lactate solution (Hartmann^®^, B. Braun Melsungen AG, Melsungen, Germany) was initiated at a rate between 3 and 5 mL kg^−1^ h^−1^. Premedication consisted of intravenous (IV) administration of dexmedetomidine (Dexdomitor^®^, Zoetis Inc., Parsippany, NJ, USA) and butorphanol (Nargesic^®^, ACME S.r.l., Cavriago, Italy) 20 min before induction of anaesthesia. Sedation was evaluated using a clinical sedation scale routinely used in the authors’ institution, as indicated in the anaesthetic record. Mild sedation was defined as slight resistance to handling, moderate sedation as full cooperation with venous catheter placement, and profound sedation as recumbency with minimal or no response to handling, allowing positioning and catheter placement without resistance.

Prior to induction, patients received pre-oxygenation via free-flow oxygen (*flow-by*) for 2–3 min. Anaesthesia was induced with IV propofol (Proposure^®^, Boehringer Ingelheim Animal Health Italia S.p.A., Milan, Italy) to effect and, after endotracheal intubation, maintained with isoflurane (IsoFlo^®^, Zoetis, Italy) delivered in a mixture of medical air and oxygen via a circle breathing system connected to an anaesthesia workstation (GE Carestation 620, GE Healthcare, Helsinki, Finland). Mechanical ventilation was provided throughout the procedure using a volume-controlled ventilation (VCV) mode to adjust end-tidal carbon dioxide concentration (ETCO_2_).

### 2.3. Monitoring and Data Collection

During anaesthesia, HR, RR, NIBP, peripheral oxygen saturation (SpO_2_), ETCO_2_, and inspired and expired anaesthetic gases (FE’ISO) were recorded every 5 min using a multiparametric monitor (Carescape B450, GE Healthcare, Helsinki, Finland). During cardiac-gated CT scan, four ECG electrodes, which were connected directly to the CT machine, were attached to the dogs’ interdigital spaces. This connection synchronizes the X-ray acquisition with the patient’s cardiac cycle. The isoflurane concentration was adjusted to maintain an adequate depth of anaesthesia, as measured by clinical signs (ventromedial rotation of the globe, reduced mandibular muscle tone, absence of voluntary movement and palpebral reflex, and lack of autonomic responses to manipulation or repositioning), cardiovascular responses (heart rate and arterial blood pressure), and FE’ISO.

During general anaesthesia, cardiovascular variables were recorded throughout the procedure. In accordance with the 2020 AAHA Anaesthesia and Monitoring Guidelines for Dogs and Cats hypotension was defined as systolic arterial blood pressure < 80–90 mmHg and MAP < 60–70 mmHg, whereas hypertension is defined as systolic arterial pressure > 160–180 mmHg and MAP > 120–140 mmHg [17]. Bradycardia and tachycardia were defined as HR < 60 beats min^−1^ and >160 beats min^−1^, respectively [19]. Any peri-anaesthetic adverse events, including hypotension, arrhythmias, or clinically relevant heart rate alterations, were recorded and treated accordingly.

Cardiac computed tomography was performed using a third-generation dual-source computed tomography scanner (192 × 2 DSCT) (Somaton Definition Force; Siemens, Erlangen, Germany). All dogs were positioned in sternal recumbency, headfirst and forelimbs extended cranially, and electrocardiographic electrodes were placed on the distal aspects of all four limbs. The ECG signal was continuously recorded and integrated into the imaging software to determine heart rate and the number of cardiac cycles during image acquisition.

An iodinated, iso-osmolar contrast medium (iodixanol; Visipaque^®^ 320 mg I mL^−1^, GE Healthcare S.r.l., Milan, Italy) was administered via the cephalic vein using a dual-syringe power injector (MEDRAD^®^ Stellant, Bayer Medical Care Inc., Indianola, PA, USA) at a flow rate of 2.0 mL s^−1^, at a total dose of 640 mg I kg^−1^ (2 mL kg^−1^), followed by an equivalent volume of isotonic saline.

Image acquisition consisted of rapid scans requiring a controlled apnoea period of approximately 15–20 s. Apnoea was achieved by mechanical hyperventilation, consisting of a transient increase in respiratory rate to reduce ETCO_2_ to 32–34 mmHg, followed by temporary cessation of ventilation immediately before the start of image acquisition.

At the end of the procedure, administration of the inhalant anaesthetic was discontinued and dogs were recovered in a quiet environment.

### 2.4. Images Evaluation

CT studies were reviewed and analyzed by a DVM with 10 years of experience in advanced diagnostic imaging (A.C.) under supervision of a DVM with 25 years of experience in advanced diagnostic imaging and PhD in Radiology (G.B).

All CT images were retrieved from the Picture Archiving Communication System (PACS) and analyzed using a dedicated stand-alone workstation and vendor-specific post-processing software (SyngoVia, Siemens, Erlangen, Germany).

Image quality was assessed by consensus using a qualitative grading system adapted from previously published studies for cardiac computed tomography in dogs [20]. Image quality was graded as *excellent*, *good*, or *moderate* based on the overall diagnostic usability of the examination.

The assessment focused on the clarity of cardiac structures and major vessels and on the presence, type, and severity of artefacts. Particular attention was given to motion-related artefacts, which have been identified as the most relevant factor affecting diagnostic quality in cardiac CT imaging. Blur of vessel margins, consistent with cardiac motion artefact, was specifically evaluated, as this represents the most frequently observed artefact in ECG-gated cardiac CT examinations in dogs.

Image quality was classified as *excellent* when cardiac anatomy and vascular structures were sharply delineated with minimal or no artefacts, allowing confident diagnostic interpretation. *Good* image quality was assigned when minor artefacts were present but did not interfere with diagnostic evaluation. *Moderate* image quality was defined by the presence of more pronounced artefacts, particularly motion-related blur, which reduced image sharpness but still allowed interpretation of the examination for clinical purposes.

### 2.5. Statistical Analysis

The free software R (http://www.r-project.org/) was used for statistical analyses. Variables were reported as mean (±SD) or median (range) values.

To evaluate the effect of premedication, heart rate (HR) measured at baseline (T00) was compared with values recorded after premedication (T0). A Student’s *t*-test was used to assess differences between the two time points.

Changes in HR, SAP, DAP, MAP, FE′ISO during anaesthesia were evaluated using the Friedman test for repeated measures. Due to the presence of missing values at later time points, the analysis was limited to the interval between T0 and 30 min after induction of anaesthesia. Mixed models were used to evaluate the presence of linear patterns in the monitored variables.

Image quality was assessed using a three-point ordinal scale (1 = excellent, 2 = good, 3 = moderate) and reported descriptively as frequencies and percentages. No inferential statistical analysis was performed due to the limited number of observations in some categories.

Statistical significance was set at *p* < 0.05.

## 3. Results

### 3.1. Study Population

Fifteen client-owned dogs (7 males and 8 females) undergoing ECG-gated CTT were included in the study. All dogs were classified as ASA physical status III or IV. Dogs represented a range of breeds, with a mean (±SD) body weight of 25 ± 17.5 kg and a mean age of 8.6 ± 3.2 years (Table 1). All patients presented with pericardial effusion, classified as mild in 10/15 dogs and moderate in 5/15 dogs, and underwent pericardial drainage prior to induction of anaesthesia.

### 3.2. Pre-Anaesthetic Echocardiographic Evaluation

Pre-anaesthetic echocardiographic evaluations revealed that 6 out of the 15 dogs (40%) exhibited mild tricuspid and/or mitral regurgitation, consistent with ACVIM stage B1 classification. Additionally, 2 dogs (13.33%) presented with moderate tricuspid and/or mitral regurgitation associated with echocardiographic signs of cardiac remodelling, classified as ACVIM stage B2 [21]. Patients presenting with cardiac tamponade were stabilised via ultrasound-guided pericardial drainage; specifically, 5 of the 15 patients underwent pericardiocentesis before premedication and 10/15 after premedication.

### 3.3. Sedation, Induction and Maintenance

Dexmedetomidine was administered at a dose of 0.67 ± 0.24 µg kg^−1^ (range 0.5–1.0 µg kg^−1^), and butorphanol at 0.24 ± 0.06 mg kg^−1^ (range 0.1–0.3 mg kg^−1^). This premedication protocol produced satisfactory sedation in all dogs. Of the 15 patients, 6 exhibited mild sedation, characterised by a calm demeanour with slight responsiveness to handling, while the remaining 9 dogs were moderately sedated, showing minimal responsiveness to external stimuli. Anaesthetic induction was achieved smoothly in all cases using intravenous propofol, with doses ranging from 2.0 to 4.5 mg kg^−1^ allowing endotracheal intubation. General anaesthesia was maintained with isoflurane at an end-tidal concentration (FE′ISO) from 0.2 to 1.5%, with a mean (±SD) of 1.10 ± 0.23% across all recorded intra-anaesthetic time points. FE′ISO varied significantly during the procedure (*p* < 0.001).

### 3.4. Cardiovascular Parameters

Heart rate (HR) ranged from 37 to 141 beats min^−1^, with a mean (±SD) of 89 ± 27 beats min^−1^, while mean arterial pressure (MAP) ranged from 58 to 117 mmHg, with a mean (±SD) of 85 ± 18 mmHg.

**Figure 1:**
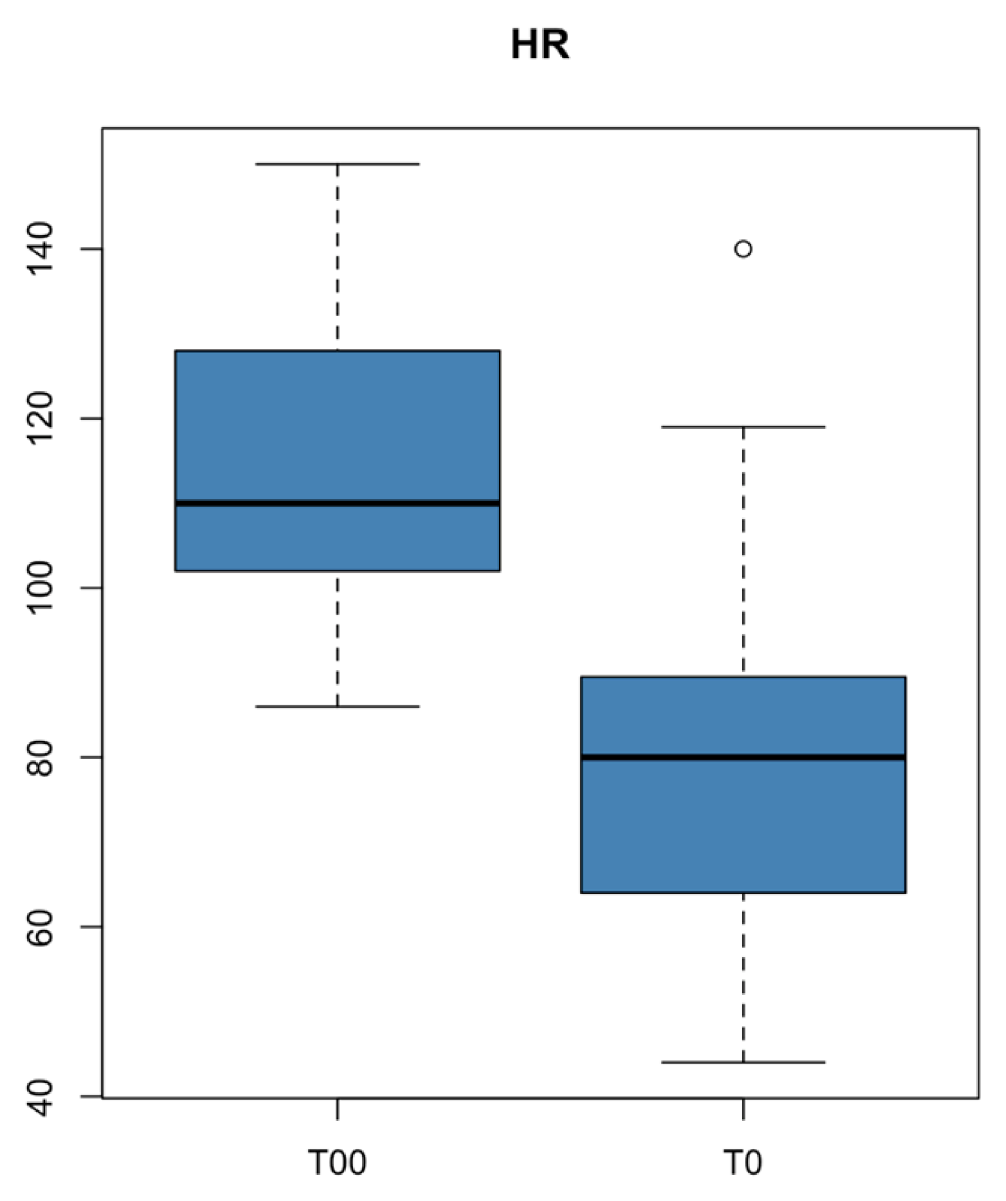
Boxplot showing the distribution of heart rate (HR) at baseline (T00) and after premedication (T0) with dexmedetomidine and butorphanol.

Comparison between baseline (T00) and post-premedication (T0) values showed a significant reduction in HR following administration of dexmedetomidine and butorphanol. HR decreased from 115 ± 16.8 beats min^−1^ at T00 to 80.6 ± 25.8 beats min^−1^ at T0 (*t* = 7.06, *p* < 0.001) as illustrated in Figure 1.

**Figure 2:**
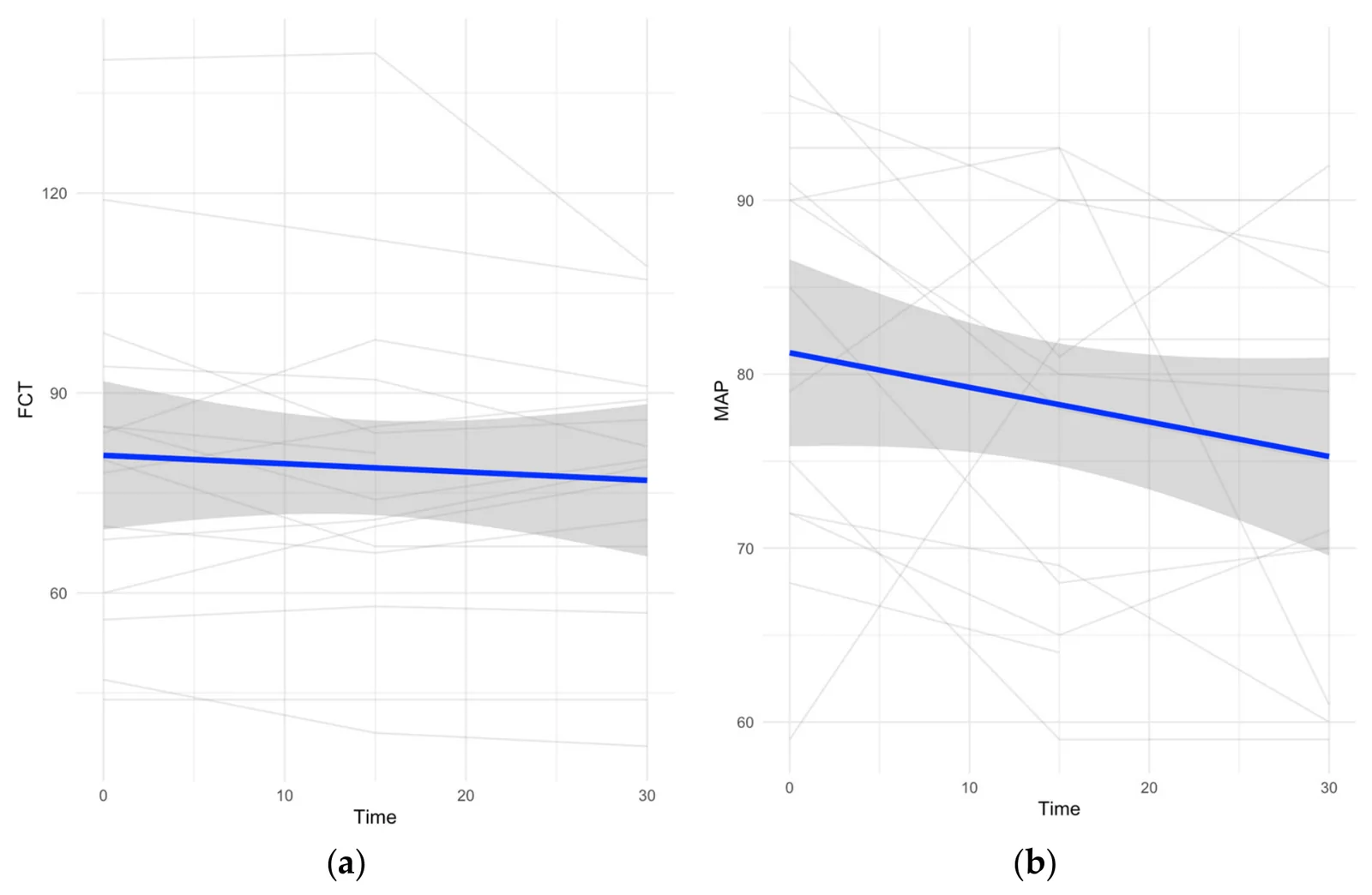
Temporal trends of (**a**) heart rate (HR) and (**b**) mean arterial pressure (MAP) during the first 30 min of anaesthesia. Data are presented as mean values at each time point. HR did not show a significant linear trend over time, whereas MAP demonstrated a mild decreasing trend.

Evaluation of cardiovascular variables during anaesthesia using the *Friedman test* revealed no significant variation in HR between T0 and 30 min (*p* = 0.722), whereas *linear mixed-effects modelling* (Figure 2) revealed a small but significant decreasing trend over time (*p* = 0.010). However, HR values remained within a relatively narrow range throughout the anaesthetic period. An exploratory analysis of temporal trends also showed a slight but significant decrease in mean and diastolic arterial pressure over time.

Mean arterial pressure MAP showed a significant variation during the first 30 min of anaesthesia (*p* = 0.024), with moderate fluctuations observed across time points.

Similarly, DAP demonstrated a significant variation over time (*p* < 0.001), whereas SAP did not show a significant change (*p* = 0.67).

**Figure 3:**
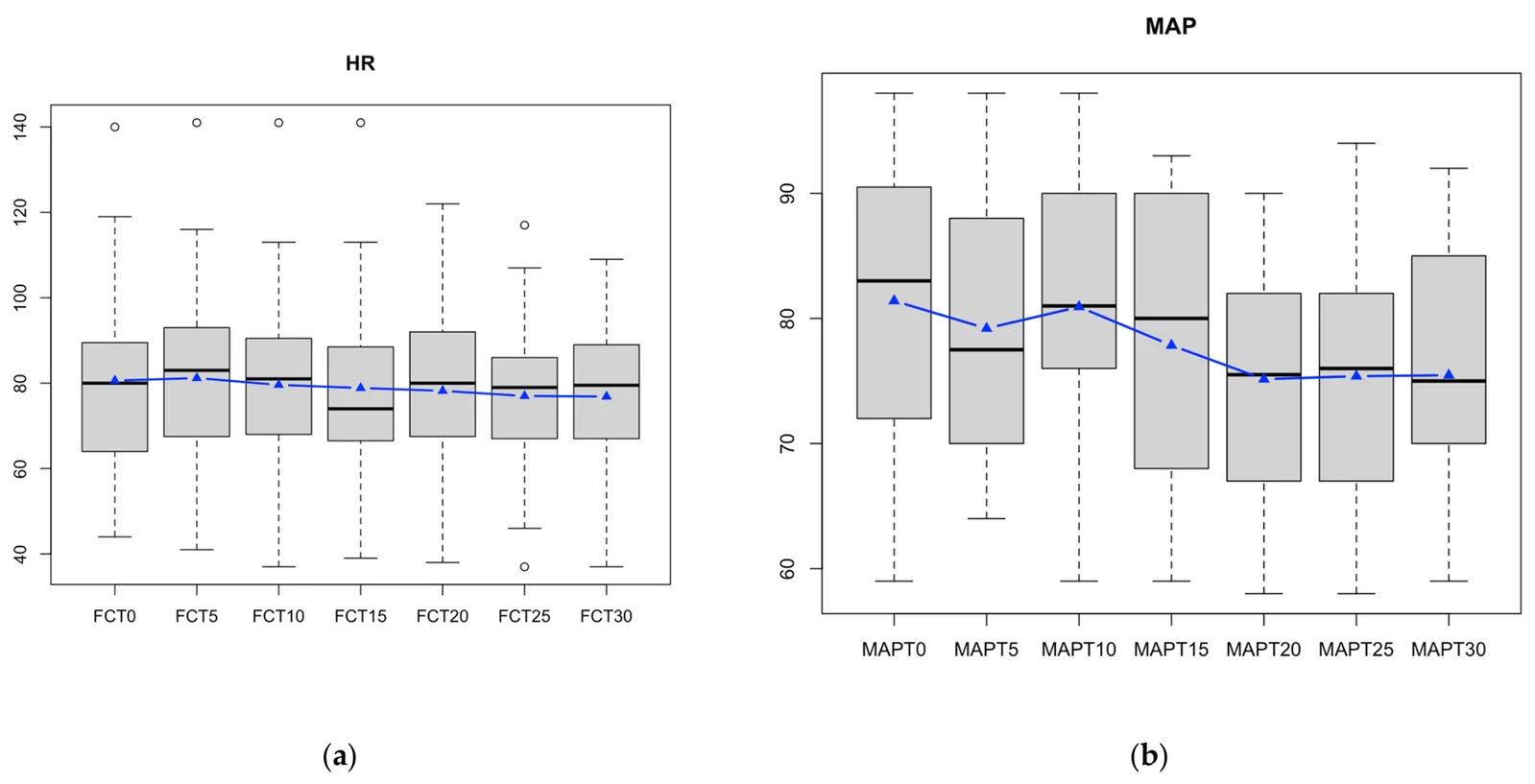
Boxplots showing the distribution of (**a**) heart rate (HR) and (**b**) mean arterial pressure (MAP) recorded at different time points during the first 30 min of anaesthesia. Boxes represent the interquartile range (IQR), the horizontal line indicates the median, whiskers represent the range of values, and dots indicate outliers. The blue dashed line with triangle markers represents the mean value at each time point, highlighting the overall temporal trend.

Two dogs experienced mild and transient hypotension, defined as MAP < 60 mmHg, which was successfully managed with intravenous crystalloid administration (Ringer’s lactate solution; Hartmann^®^, B. Braun Vet Care, Melsungen, Germany) at 5–10 mL kg^−1^ over 15–20 min. Importantly, no arrhythmias were recorded during the procedure. Temporal trends of HR and MAP are shown in Figure 2. The distribution of HR and MAP values is illustrated in Figure 3.

### 3.5. Ventilatory Parameters

Mechanical ventilation was delivered using a volume-controlled mode in all dogs. Tidal volume normalised to body weight ranged from 7.0 to 15.0 mL kg^−1^, with a mean (±SD) of 10.5 ± 1.7 mL kg^−1^ during the procedure. Ventilation was adjusted to maintain normocapnia throughout anaesthesia and to ensure apnoea during selected CT phases.

### 3.6. Anaesthesia Duration and Recovery

Anaesthesia duration averaged 43 ± 12.5 min, with extubation occurring 7 ± 4.7 min after discontinuation of isoflurane administration. No adverse events or mortality were recorded within 48 h following the procedure.

### 3.7. Image Quality Assessment

**Figure 4:**
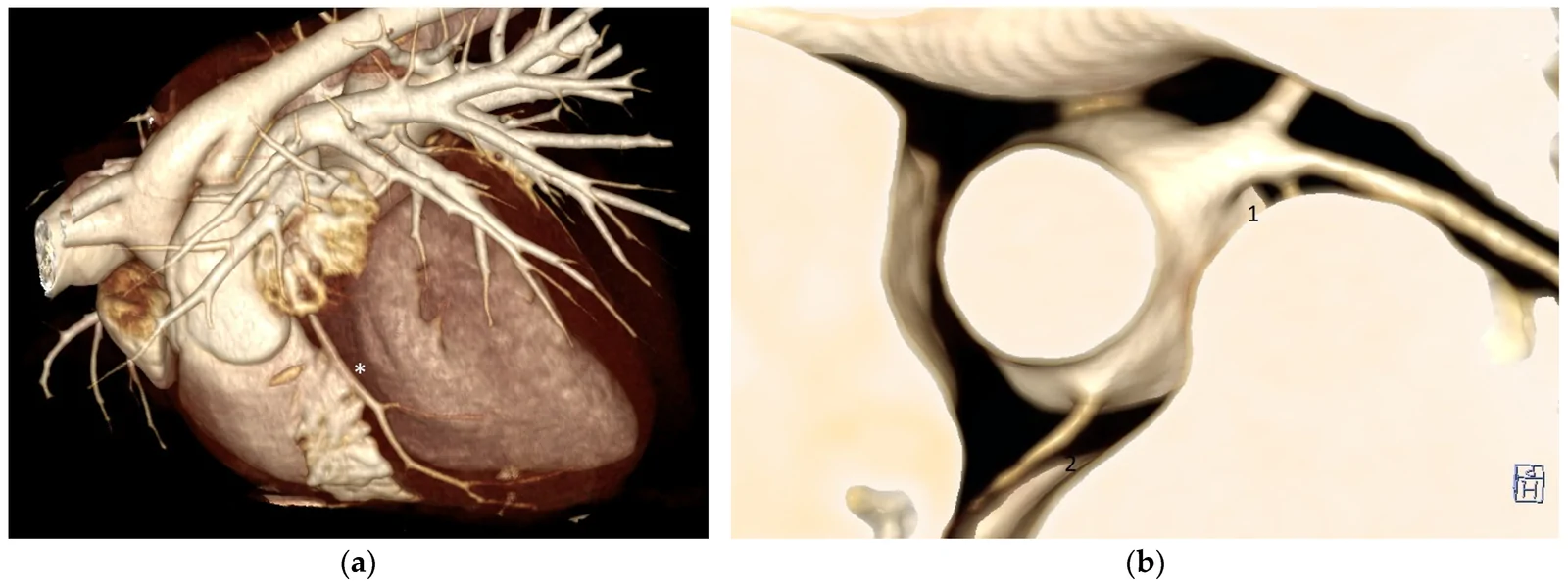
Representative volume-rendered cardiac CT images. (**a**) Lateral view showing the paraconal interventricular branch of the left coronary artery (*). (**b**) Transverse view showing the left (1) and right coronary (2) arteries.

The cardiac images acquired during this study were thoroughly analysed, allowing for the diagnosis of underlying pathological conditions in all animals examined. Representative volume-rendered cardiac CT images illustrating coronary artery visualisation are shown in Figure 4. In the majority of cases (10/15, 66.67%), a cardiac neoplasm was identified, including atrial tumours (6/15), tumours at the base of the heart (3/15), and a ventricular mass (1/15). In the remaining cases (5/15, 33.33%), a diagnosis of pericardial disease was made.

**Figure 5:**
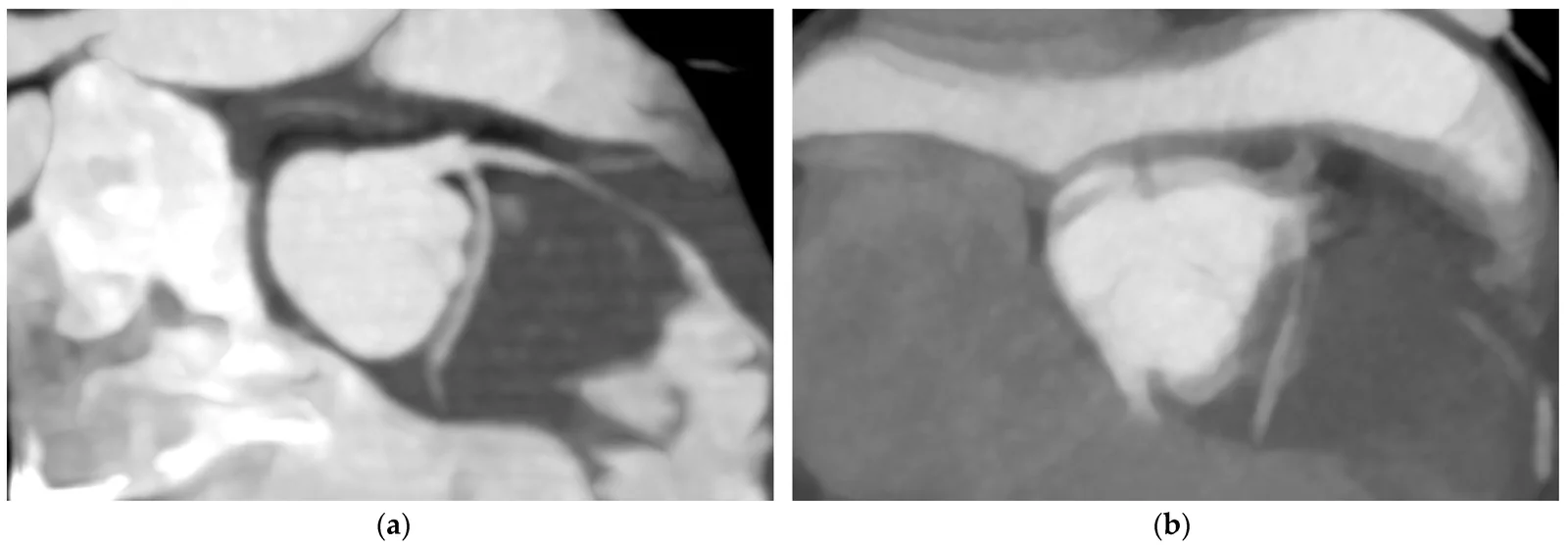
Representative transverse cardiac CT images. (**a**) Diagnostic-quality image showing the left coronary artery and its branches. (**b**) Image affected by motion artefact, preventing visualization of the left coronary artery.

**Figure 6:**
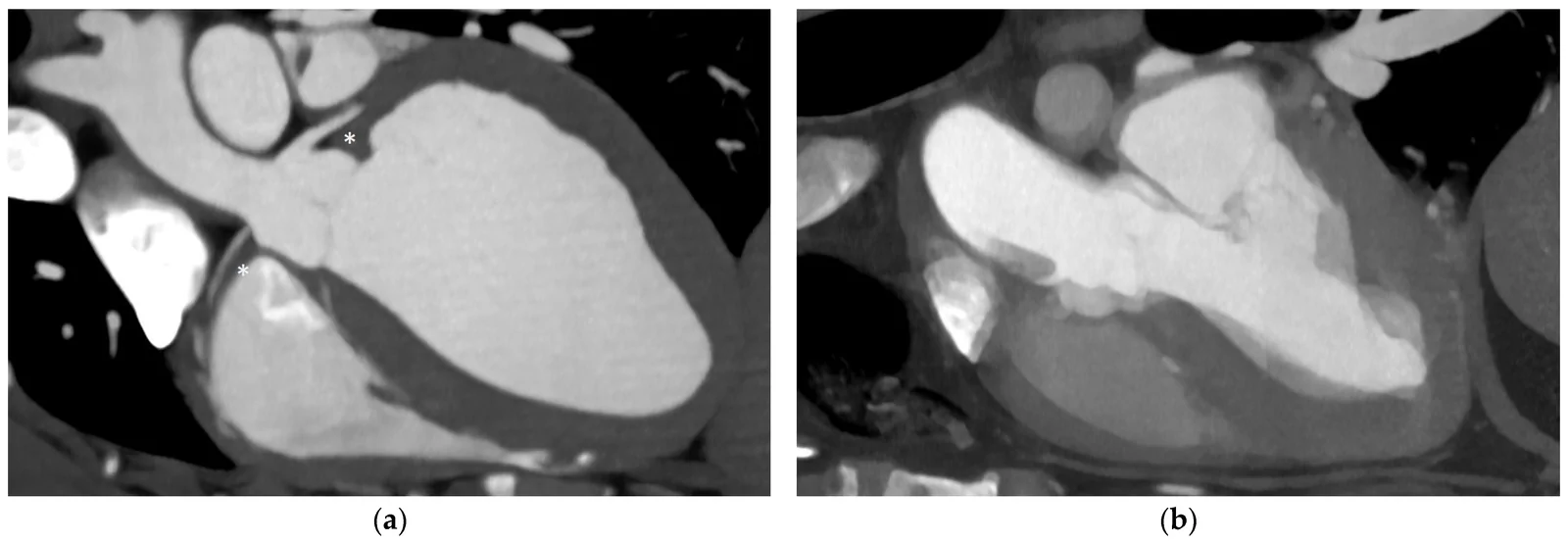
Representative lateral cardiac CT images illustrating differences in image quality. (**a**) Diagnostic-quality image in which the left coronary arteries are visible (*). (**b**) Image affected by motion-related artefacts.

The quality of imaging was categorised as excellent in 46.67% of cases (7/15) and good in 40% (6/15). In two cases (13.33%), the image quality was rated as moderate due to the presence of artefacts, one of which was attributed to movement despite ensuring patient immobilisation and apnoea. Representative transverse and lateral cardiac CT images illustrating diagnostic image quality and motion-related artefacts are shown in Figure 5 and Figure 6.

## 4. Discussion

This case series suggests that low-dose dexmedetomidine may represent a feasible component of an anaesthetic protocol for dogs with cardiac disease undergoing ECG-gated cardiac computed tomography (CCT). The protocol provided clinically useful sedation while preserving haemodynamic stability during general anaesthesia, a key requirement in high-risk cardiopathic patients. Given the retrospective nature of this case series, these findings should be interpreted descriptively rather than as evidence of causal relationships. Previous studies have described the dose-dependent cardiovascular effects of dexmedetomidine, whereby low doses may provide sedative benefits while having limited impact on cardiac output and arterial blood pressure [6,22]. The sympatholytic and mild analgesic properties of dexmedetomidine, combined with butorphanol, may have contributed to this stability by attenuating stress-related sympathetic activation and facilitating heart rate control [22]. A relevant finding of this study was the high diagnostic quality of the cardiac CT examinations. The majority of scans were graded as excellent or good, indicating that the combination of the anaesthetic protocol and the imaging conditions was effective in producing diagnostically reliable images. The high performance and efficiency of the specific CT scanner used in this study likely played a significant role in achieving this level of image quality. The high acquisition speed and temporal resolution of this system allowed rapid data collection during ECG-gated acquisitions, thereby reducing susceptibility to motion artefacts and enhancing overall image clarity, even in dogs with underlying cardiac disease. Many of these masses detected with the CT scan were noticed during prior echocardiographic examinations.

Premedication with dexmedetomidine and butorphanol may have further contributed to image quality by facilitating sedation and heart rate control, thereby potentially reducing motion artefacts during ECG-gated acquisitions. Previous studies have demonstrated that lower and more stable heart rates improve image consistency and reduce motion-related degradation in cardiac CT examinations [23]. In the present case series, this effect was particularly relevant given the need for precise structural assessment of the heart in dogs with pericardial disease. As shown in Figure 5 and Figure 6, when image quality was adequate, cardiac and coronary structures, including the left and right coronary arteries and their branches, could be clearly identified, whereas motion-related artefacts reduced image sharpness and impaired differentiation of small coronary structures.

The two examinations graded as having moderate image quality occurred in dogs with significant residual pericardial effusion despite prior drainage, which likely contributed to subtle residual cardiac motion despite appropriate immobilisation and apnoea. This specific case of motion artefact was therefore more likely related to the underlying pathological condition rather than to limitations of either the anaesthetic protocol or the imaging system itself. This observation supports the importance of adequate preprocedural stabilisation, including pericardial drainage when indicated, to optimise image quality in ECG-gated cardiac CT. In another study, lower image quality scores were predominantly observed in distal coronary segments, while proximal segments more frequently achieved higher diagnostic scores, partly due to increased blurring in distal vessels [20]. The application of a structured image quality scoring system provided a standardised framework for assessing diagnostic usability and highlighted the importance of minimising patient motion, particularly in dogs with pericardial effusion. Sedation quality was clinically satisfactory in all dogs, with sedation consistently categorised as mild or moderate. Moderate sedation, observed in the majority of patients, facilitated venous catheter placement and smooth induction of anaesthesia while preserving haemodynamic stability. Recent recommendations in dogs with myxomatous mitral valve disease (MMVD), suggest that low doses of α2-agonists, such as dexmedetomidine 0.5–1 μg/kg IV or 2–3 μg/kg IM, may be considered for sedation in selected nervous dogs with ACVIM stage B1 disease [24]. During anaesthesia, dexmedetomidine’s cardiovascular effects, particularly the reduction in heart rate and increase in blood pressure, align with findings from previous studies on dexmedetomidine and medetomidine sedation protocols, which showed similar trends in hemodynamic responses [25].

Induction with propofol has been associated with a transient reduction in arterial blood pressure in dogs, while cardiac output may be preserved through compensatory chronotropic responses, as reported in healthy animals using advanced haemodynamic monitoring [26]. This interpretation is supported by a recent prospective clinical study in healthy dogs, in which low-dose dexmedetomidine improved preoperative sedation, reduced propofol requirements for induction, and was not associated with significant differences in intraoperative heart rate or arterial blood pressure compared with a protocol without dexmedetomidine [27]. In the present case series, titration of propofol to effect, combined with premedication and close cardiovascular monitoring, allowed smooth induction without clinically relevant adverse events in dogs suffering from cardiac disease. Moreover, the anaesthetic protocol used appeared to ensure haemodynamic stability, as the vital parameters remained within normal limits. It must be remembered that cardiac output was not clinically monitored; therefore, information about actual perfusion, and consequently oxygen delivery, is lacking.

Several limitations of this study should be acknowledged. The retrospective design and limited sample size restrict the ability to control for confounding variables. The absence of a control group prevents assessment of the specific contribution of dexmedetomidine, butorphanol, or other components of the anaesthetic protocol and precludes conclusions regarding the safety or efficacy of the protocol. Dexmedetomidine and butorphanol were administered within clinical dose ranges but were not standardised across all patients, which may have contributed to variability in haemodynamic responses, thereby precluding precise dose–response evaluation. Furthermore, because only dogs managed with this particular protocol were included, selection bias cannot be excluded. It remains possible that dogs considered more haemodynamically unstable were managed using alternative anaesthetic approaches and were therefore not represented in the present cohort. Dexmedetomidine and butorphanol were administered within clinical dose ranges but were not standardised across all patients, which may have contributed to variability in haemodynamic responses and precludes precise dose–response evaluation. Another limitation of this study is the lack of invasive blood pressure measurement and direct cardiac output assessment. Haemodynamic stability was inferred from indirect parameters such as heart rate and non-invasive arterial blood pressure (NIBP), which may not fully reflect changes in cardiac performance. Echocardiographic studies in healthy dogs sedated with dexmedetomidine in combination with butorphanol have demonstrated measurable effects on cardiac function despite clinically acceptable cardiovascular variables, highlighting the limitations of relying solely on conventional haemodynamic monitoring [25,28].

Finally, while image quality and sedation outcomes were favourable, standardised and widely accepted scoring systems for sedation depth and motion artefacts in veterinary cardiac imaging remain limited. The development of validated assessment tools would enhance comparability between studies and facilitate the optimisation of anaesthetic protocols for high-risk patients. Overall, the present case series suggests that this anaesthetic protocol, in combination with the use of a high-performance cardiac CT system and appropriate patient stabilisation, may represent a useful approach for achieving diagnostically useful cardiac imaging in dogs with cardiac disease.

## 5. Conclusions

This case series suggests that the described anaesthetic protocol can be feasible for use in dogs with cardiac disease undergoing ECG-gated CCT. Recorded cardiovascular variables remained within clinically acceptable ranges in most dogs, and all examinations produced diagnostically interpretable images. While the findings are promising, further research with larger sample sizes and prospective designs is recommended to confirm the safety and efficacy of this protocol in broader clinical settings. Future studies could also compare dexmedetomidine with alternative sedatives to refine guidelines for anaesthetic protocols tailored specifically to cardiopathic dogs undergoing imaging procedures. Overall, this study contributes valuable insights into the management of high-risk anaesthesia in veterinary cardiology, offering a protocol that balances sedation, cardiovascular stability, and diagnostic quality.

## Figures and Tables

**Table 1:** Signalment and clinical characteristics of the 15 dogs included in the study. Age is reported in months. Body condition score (BCS) was assessed on a 5-point scale. ASA physical status classification was assigned according to the American Society of Anesthesiologists criteria.

Age (Months)	Weight (kg)	Sex	BCS (/5)	ASA	Breed
108	29.4	FI	3/5	III	Golden Retriever
73.2	29.6	FO	3/5	III	Siberian Husky
134.4	28	MI	3/5	III	British bulldog
115.2	30.7	FO	3/5	III	Golden Retriever
50.4	53	MI	4/5	III	Golden Retriever
135.6	28.3	FO	3/5	III	Siberian Husky
63.6	11.1	MI	3/5	III	Boston Terrier
108	10.6	FO	3/5	III	French bulldog
168	10.8	FO	2/5	III	Cocker Spaniel
132	38.8	FO	3/5	III	Argentine Mastiff
85.2	8.3	MC	3/5	III	Mixed Breed
84	9.8	MC	4/5	IV	Pug
25.2	64.9	MI	3/5	III	Neapolitan Mastiff
130.8	13.9	MI	3/5	III	French bulldog
136.8	13	FO	3/5	III	Mixed Breed

FI = female intact; FO = female ovariectomised; MI = male intact; MC = male castrated; BCS = body condition score; ASA = American Society of Anesthesiologists physical status.

## Data Availability

The data presented in this study are available on reasonable request from the corresponding author. In accordance with the policy of the authors’ institution, datasets related to institutional research activities are made available upon request rather than through direct public deposition. Data may be shared with qualified researchers, subject to institutional approval and in compliance with applicable ethical, privacy, and data governance requirements.

## Abbreviations

The following abbreviations are used in this manuscript:

ACVIM	American College of Veterinary Internal Medicine
ASA	American Society of Anaesthesiologists
BP	Blood pressure
CCT	Cardiac computed tomography
CT	Computed tomography
ECG	Electrocardiography
ECG-GCT	Electrocardiography-gated computed tomography
ETCO_2_	End-tidal carbon dioxide
FE′ISO	End-tidal isoflurane concentration
HR	Heart rate
IV	Intravenous
MAP	Mean arterial pressure
NIBP	Non-invasive blood pressure
RR	Respiratory rate
SpO_2_	Peripheral oxygen saturation
VCV	Volume-controlled ventilation

## References

[1] Scollan K.F., Bottorff B., Stieger-Vanegas S., Nemanic S., Sisson D. (2015). Use of multidetector computed tomography in the assessment of dogs with pericardial effusion. J. Vet. Intern. Med..

[2] Bertolini G., Angeloni L., Halefoglu A.M. (2017). Vascular and cardiac CT in small animals. Computed Tomography—Advanced Applications.

[3] Kim J., Lee H., Hwang J., Yoon J. (2023). Clinical utility of a new protocol of cardiac computed tomography in dogs. Vet. Med. Sci..

[4] Bertolini G. (2017). Body MDCT in Small Animals: Basic Principles, Technology, and Clinical Applications.

[5] Pan S.-Y., Liu G., Lin J.-H., Jin Y.-P. (2021). Efficacy and safety of dexmedetomidine premedication in balanced anesthesia: A systematic review and meta-analysis in dogs. Animals.

[6] Murrell J.C., Hellebrekers L.J. (2005). Medetomidine and dexmedetomidine: A review of cardiovascular effects and antinociceptive properties in the dog. Vet. Anaesth. Analg..

[7] Mason K.P., Zgleszewski S.E., Prescilla R., Fontaine P.J., Zurakowski D. (2008). Hemodynamic effects of dexmedetomidine sedation for CT imaging studies. Paediatr. Anaesth..

[8] Alotaibi N.S. (2024). Pediatric sedation outside the operating room integrating dexmedetomidine for MRI and CT scan procedures: A systematic review. Saudi J. Anaesth..

[9] Koroglu A., Demirbilek S., Teksan H., Sagir O., But A.K., Ersoy M.O. (2005). Sedative, haemodynamic and respiratory effects of dexmedetomidine in children undergoing magnetic resonance imaging examination. Br. J. Anaesth..

[10] Takahashi K., Yoshikawa Y., Kanda M., Hirata N., Yamakage M. (2023). Dexmedetomidine as a cardioprotective drug: A narrative review. J. Anesth..

[11] Liu Y., Chen Q., Hu T., Deng C., Huang J. (2024). Dexmedetomidine administration is associated with improved outcomes in critically ill patients with acute myocardial infarction partly through its anti-inflammatory activity. Front. Pharmacol..

[12] Kuusela E., Raekallio M., Anttila M., Falck I., Mölsä S., Vainio O. (2002). Clinical effects and pharmacokinetics of medetomidine and its enantiomers in dogs. J. Vet. Pharmacol. Ther..

[13] Wang H.-C., Hung C.-T., Lee W.-M., Chang K.-M., Chen K.-S. (2016). Effects of intravenous dexmedetomidine on cardiac characteristics measured using radiography and echocardiography in healthy dogs. Vet. Radiol. Ultrasound.

[14] Willigers H.M., Prinzen F.W., Roekaerts P.M.H.J., de Lange S., Durieux M.E. (2003). Dexmedetomidine decreases perioperative myocardial lactate release in dogs. Anesth. Analg..

[15] Martin-Flores M., Moy-Trigilio K.E., Campoy L., Araos J. (2021). The use of dexmedetomidine during pulmonic balloon valvuloplasty in dogs. Vet. Rec..

[16] da Cunha A.F., Ramos S.J., Domingues M., Beaufrère H., Shelby A., Stout R., Acierno M.J. (2016). Agreement between two oscillometric blood pressure technologies and invasively measured arterial pressure in the dog. Vet. Anaesth. Analg..

[17] Grubb T., Sager J., Gaynor J.S., Montgomery E., Parker J.A., Shafford H., Tearney C. (2020). 2020 AAHA anesthesia and monitoring guidelines for dogs and cats. J. Am. Anim. Hosp. Assoc..

[18] American Society of Anesthesiologists (2026). American Society of Anesthesiologists Statement on ASA Physical Status Classification System. Anesthesiol. Open.

[19] Haskins S.C., Grimm K.A., Lamont L.A., Tranquilli W.J., Greene S.A., Robertson S.A. (2024). Monitoring anesthetized patients. Veterinary Anesthesia and Analgesia.

[20] Drees R., Johnson R.A., Pinkerton M., Muñoz del Rio A., Saunders J.H., François C.J. (2015). Evaluating the effect of two different anesthetic protocols on 64-MDCT coronary angiography in dogs. Vet. Radiol. Ultrasound.

[21] Atkins C., Bonagura J., Ettinger S., Fox P., Gordon S., Häggström J., Hamlin R., Keene B., Luis Fuentes V., Stepien R. (2009). Guidelines for the diagnosis and treatment of canine chronic valvular heart disease. J. Vet. Intern. Med..

[22] Di Franco C., Evangelista F., Briganti A. (2023). Multiple uses of dexmedetomidine in small animals: A mini review. Front. Vet. Sci..

[23] Kalisz K., Buethe J., Saboo S.S., Abbara S., Halliburton S., Rajiah P. (2016). Artifacts at Cardiac CT: Physics and Solutions. RadioGraphics.

[24] Levinzon I., Köster L., Vettorato E., Estrada A.H. (2026). Pre-anaesthetic risk assessment and management of dogs with myxomatous mitral valve disease: A spectrum of care narrative review. J. Small Anim. Pract..

[25] Kellihan H.B., Stepien R.L., Hassen K.M., Smith L.J. (2015). Sedative and echocardiographic effects of dexmedetomidine combined with butorphanol in healthy dogs. J. Vet. Cardiol..

[26] Cattai A., Rabozzi R., Ferasin H., Isola M., Franci P. (2018). Haemodynamic changes during propofol induction in dogs: New findings and approach of monitoring. BMC Vet. Res..

[27] Klasič M., Seliškar A., Rakinić K., Ipavec M., Sredenšek J., Tomsič K. (2025). Sedative and cardiovascular effects of premedication with acepromazine, methadone and dexmedetomidine in sevoflurane-anaesthetized dogs. Vet. Anaesth. Analg..

[28] Bočkay A., Agudelo C.F., Figurová M., Vargová N., Trbolová A. (2024). Effect of Butorphanol-Medetomidine and Butorphanol-Dexmedetomidine on Echocardiographic Parameters during Propofol Anaesthesia in Dogs. Animals.

